# A novel method for periapical microsurgery with the aid of 3D technology: a case report

**DOI:** 10.1186/s12903-018-0546-y

**Published:** 2018-05-10

**Authors:** Shangzhu Ye, Shiyong Zhao, Weidong Wang, Qianzhou Jiang, Xuechao Yang

**Affiliations:** 1 Department of Digital Dental Center, Key Laboratory of Oral Medicine, Guangzhou Institute of Oral Disease, Stomatology Hospital of Guangzhou Medical University, 59 Huangsha Road, Guangzhou, 510140 Guangdong Province China; 2 Department of Endodontics, Key Laboratory of Oral Medicine, Guangzhou Institute of Oral Disease, Stomatology Hospital of Guangzhou Medical University, 39 Huangsha Road, Guangzhou, Guangdong Province China

**Keywords:** 3D printing technology, Endodontic microsurgery, Guided periapical surgery, Root-end resection

## Abstract

**Background:**

Three-dimensional (3D) technology has gained wide acceptance in dentistry. It has been used for treatment planning and surgical guidance. This case report presented a novel treatment approach to remove cortical bone and root-end during periapical surgery with the help of Cone-Beam Computed Tomography (CBCT), Computer Aided Design (CAD) and three-dimensional (3D) printing technology.

**Case presentation:**

A 37-year-old female patient presented with a large periapical lesion of left maxillary lateral incisor and canine was referred for microsurgical endodontic surgery. The data acquired from a preoperative diagnostic CBCT scan and an intra-oral scan was uploaded into surgical planning software and matched. A template that could be used to locate root-ends and lesion areas was virtually designed based on the data and was fabricated using a 3D printer. With the guidance of the template, the overlying cortical bone and root-end were precisely removed by utilizing a trephine with an external diameter of 4.0 mm. The patient was clinically asymptomatic at a six-month follow-up review. One year after the surgery, the lesion was healing well and no periapical radiolucency was observed on radiographic examination.

**Conclusions:**

The digitally designed directional template worked in all aspects to facilitate the periapical surgery as anticipated. The root-ends were accurately located and resected. The surgical procedure was simplified, and the treatment efficiency was improved. This technique minimized the damage and reduced iatrogenic injury.

## Background

When a radiotransparent periapical lesion measures over 8 to 10 mm in diameter [1] and it is a suspected periapical cyst, endodontic surgery is required to remove the cyst and a biopsy is needed to confirm histologic diagnosis of the lesion [2].

A most commonly performed endodontic surgery usually involves exposure of the periapical lesion through an osteotomy, surgical removal of the lesion, removal of part of the root-end tip [3]. However, the root-end surface sometimes can be difficult to distinguish from the surrounding osseous tissues [4].

In such cases, conventionally, the approximate location of the root-end may be estimated using preoperative radiographs [3]. The method of locating the root apex is to first locate the body of the root substantially coronal to the apex, where the bone covering the root is thinner. Once the root has been located and identified, the bone covering the root is slowly and carefully removed, working in an apical direction until the root apex is identified [5].

The limitations and disadvantages of the classical surgical method have become apparent due to the rapid advance of technology. Firstly, searching for root apex from the coronal direction of root end inevitably increases damage and risk to non-pathological osseous tissues [4]. Secondly, conventional radiography shows only two-dimensional images, which does not represent the lesion area accurately and distinctly. Thirdly, it is not easy for inexperienced endodontic surgeons to balance between limiting damage to osseous tissues and gaining enough visual and operative access for root-end resection and root-end filling [4]. Based on CBCT, CAD and 3D printing technology, however, these problems can be solved.

Rapid prototyping technology, better known as 3D printing, has provided new possibilities for diagnosis, surgical planning, prosthesis design, and student education in medicine. In dentistry, 3D printing technology has been used for treatment planning, surgical guidance, and the fabrication of dental models for appliances in orthognathic surgery, implant surgery, oral and maxillofacial surgery, orthodontics, and prosthodontics [6–10]. The high accuracy of 3D printing and extended flexibility render this technology very promising [11–15]. In endodontics, 3D printing has also gained wide application. Kim et al. [16] fabricated a 3D printed physical tooth model to aid the endodontic treatment of an anomalous anterior tooth; a 3D printed template was used in root canal treatment for teeth with pulp canal calcification [17], and its accuracy was proven [11]; Shi X et al. [18] described the application of a 3D printed template for the predictable navigation of obliterated canal systems during root canal treatment to avoid iatrogenic damage of the root [18]; In endodontic surgery, a 3D printed retractor was invented and fabricated for soft tissue retraction [8].

This case report describes a novel method for guided periapical surgery, which removed overlying cortical bone and root-end precisely with the aid of a 3D-printed surgical template.

## Case Presentation

A 37-year-old female patient presented with discomfort in the left maxillary lateral incisor. Clinical examination revealed that the left maxillary lateral incisor and canine were slightly tender to percussion. Pulp vitality test showed a negative response to temperature for both teeth. Radiograph showed a large periapical radiolucency around both teeth. The patient was clinically diagnosed with chronic periapical periodontitis. Considering the large size of the periapical lesion and it was a suspected periapical cyst, we decided to treat the patient with a microsurgical endodontic surgery for biopsy and to remove the root-ends at the same time to eliminate contamination. No contraindications were found. The patient had no significant medical history and was in good medical status.

**Fig. 1:**
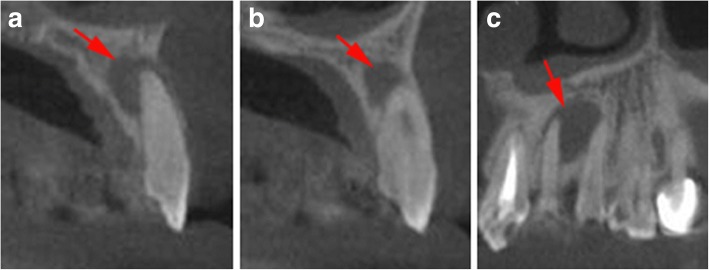
**a**, **b** Sagittal CBCT images of the left maxillary lateral incisor and canine showed lesions in periradicular regions. **c** An oblique coronal CBCT image revealed that the teeth shared one elliptic lesion with an approximate size of 13 mm*9 mm*9 mm. The red arrow indicates the location of the lesion

After obtaining the patient’s informed consent about the surgery procedure and the possible prognosis of the outcome, a small volume CBCT scan (iCAT 17–19, Imaging Sciences International, Hatfield, PA, USA) was taken to obtain a more detailed view of the periapical area, to determine the accurate size of the lesion and the exact location of root apices, to evaluate the proximity of adjacent anatomical structures and to design a template. A well-defined radiolucent lesion with an approximate size of 13 mm*9 mm*9 mm at the apices of the upper left lateral incisor and canine was observed on CBCT (Fig. 1). An endodontic specialist treated her with an appropriate root canal therapy before the surgery.

**Fig. 2:**
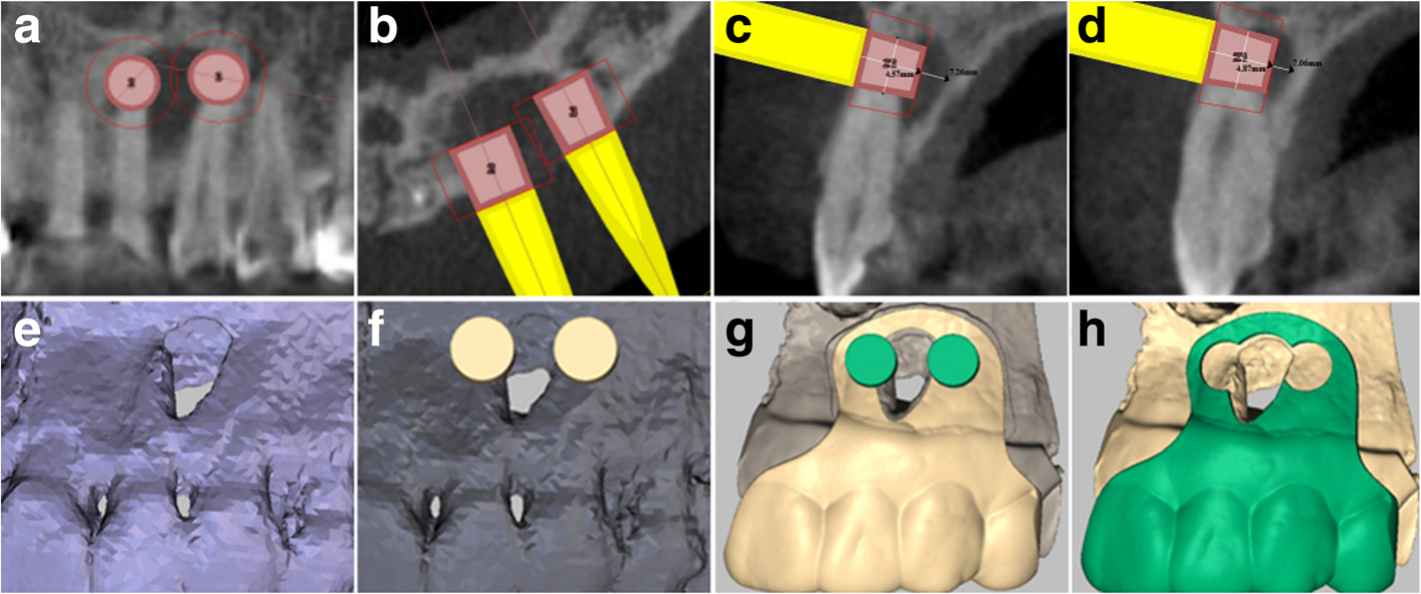
**a** A 3 mm root-end for resection was marked by a simulated virtual trephine with a diameter of 4 mm on an oblique coronal section of the left maxillary lateral incisor and canine. The root-ends of the left maxillary central incisor and first premolar were safe from accidental damage. **b** A horizontal section indicated the location of root-ends for both teeth and the root-ends were marked by the simulated virtual trephine. **c** Based on a sagittal section of the left maxillary lateral incisor, we learned that a working depth of 4.57 mm would be sufficient for the trephine to remove the root-end completely. This depth was still 2.69 mm away from the palatal cortical bone, which we regarded as a safe depth. **d** For the left maxillary canine, the working depth was 4.87 mm, and the safe depth was 2.19 mm. All lengths were measured using a tool provided by the software. **e** Three-dimensional reconstruction of the scans obtained from CBCT and surface scans were matched to reconstruct the operating site. **f** The locations of the root-ends of the left maxillary lateral incisor and canine were marked out on the reconstruction image. **g** The template was designed to be supported by teeth from the left maxillary central incisor to the left maxillary first premolar. The lesion area was located, and the outline was confirmed. **h** The template was designed to be 2 mm thick after considering the flexural strength of the resin composite. A 0.5 mm space from the labial cortical plate to the template was preserved to accommodate soft tissues

The acquired Digital Imaging and Communications in Medicine (DICOM) files from the CBCT images were uploaded into a software (Simplant, Leuven Belgium) for virtual surgical planning. A digital impression was acquired with an intra-oral scanner (3Shape, Denmark) and uploaded into the same software. Both the CBCT and the surface scan were matched based on radiographically visible teeth. A template was virtually designed to locate the lesion area and the root apex of the teeth precisely (Fig. 2a and b). The thickness of labial cortical bone was gauged using a virtual measure tool provided by the software and recorded as working depth I. The straight distance from the surface of the labial cortical bone to the palatal side of the root-end requiring resection was also gauged and recorded as working depth II. The distance from the palatal side of the root-end to the labial side of the palatal alveolar bone was also gauged and recorded as safe depth to prevent the trephine from entering too deeply into the bone and causing unnecessary damage to the palatal alveolar bone (Fig. 2c and d). During the surgery, the thickness of the template (2.0 mm) and the space reserved for soft tissue (0.5 mm) was added to the working depth I and II to obtain total working depth I and II.

**Fig. 3:**
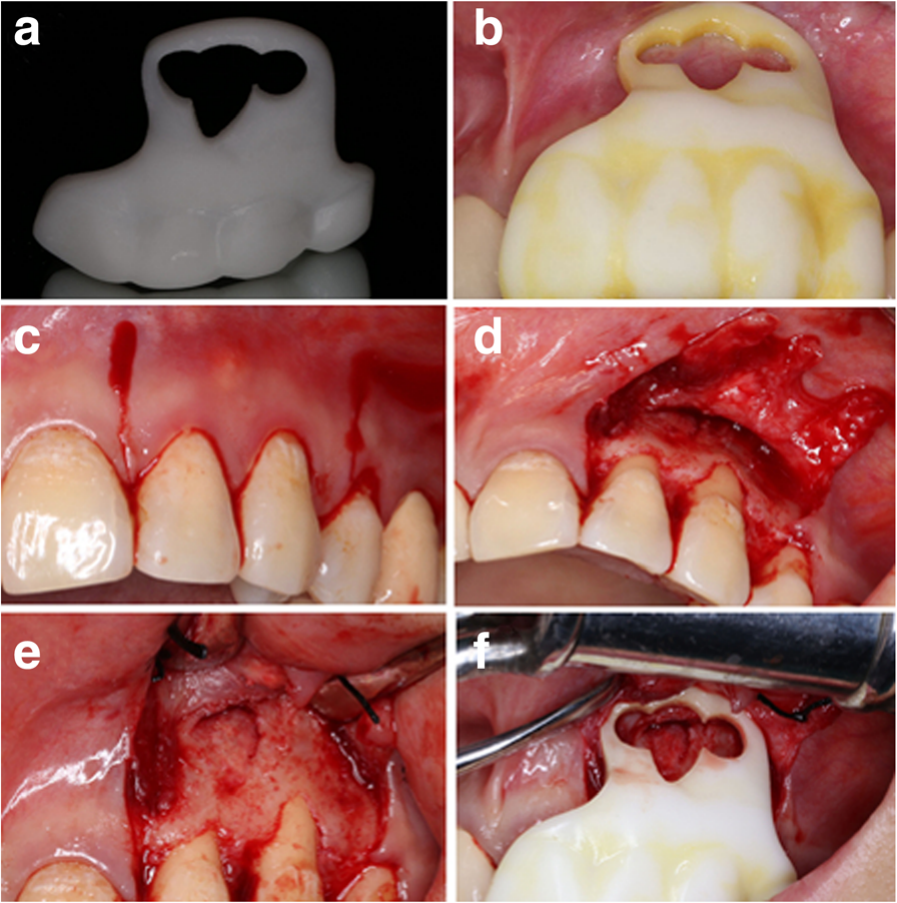
**a** The template was fabricated exactly as designed with an equivalent thickness. **b** The disinfected template was positioned on the real teeth and checked. **c** A full-thickness marginal flap was prepared with a primary incision in the gingival sulcus and the relieving incision as vertical as possible to avoid severing supra-periosteal vessels and collagen fibres. **d** The reflection of the flap and the exposure of the lesion. **e** Sling suture to the buccal mucosa. **f** The template was placed in position and checked

To guide and accommodate a trephine (Meisinger, Germany) with an external diameter of 4.0 mm, the round hollow part aimed to locate the root-end was designed with a diameter of 4.2 mm, which was enough to hold the trephine but not so large as to destroy accuracy. To preserve more root length and avoid exposing more dentinal tubules, the track guiding the trephine was designed to be perpendicular to the long axis of the root. The other hollow part lying in the middle followed the outline of the lesion, locating the whole part of the lesion precisely (Fig. 2e, f, g and h). The virtual template was exported as a stereolithography (STL) file and was fabricated using a 3D printer (3510SD, 3D system Corporation, Rock Hills, SC, USA) (Fig. 3a).

After fabrication, the template was positioned on the patient’s plaster cast, and its correct and reproducible fitting was checked. It was then detached and soaked in disinfectant for use. Another fitness check was performed on the real teeth of the patient before surgery (Fig. 3b).

After disinfection of the skin and mucosa, primacaine was delivered into the loose connective tissue of the alveolar mucosa near the root apices for local anaesthesia. A rectangular, full-thickness flap design was chosen in this case (Fig. 3c). The mucoperiosteum was reflected, and the labial alveolar plate was exposed where a semi-lunar perforation was observed (Fig. 3d). We sutured the flap to the labial mucosa (Fig. 3e).

**Fig. 4:**
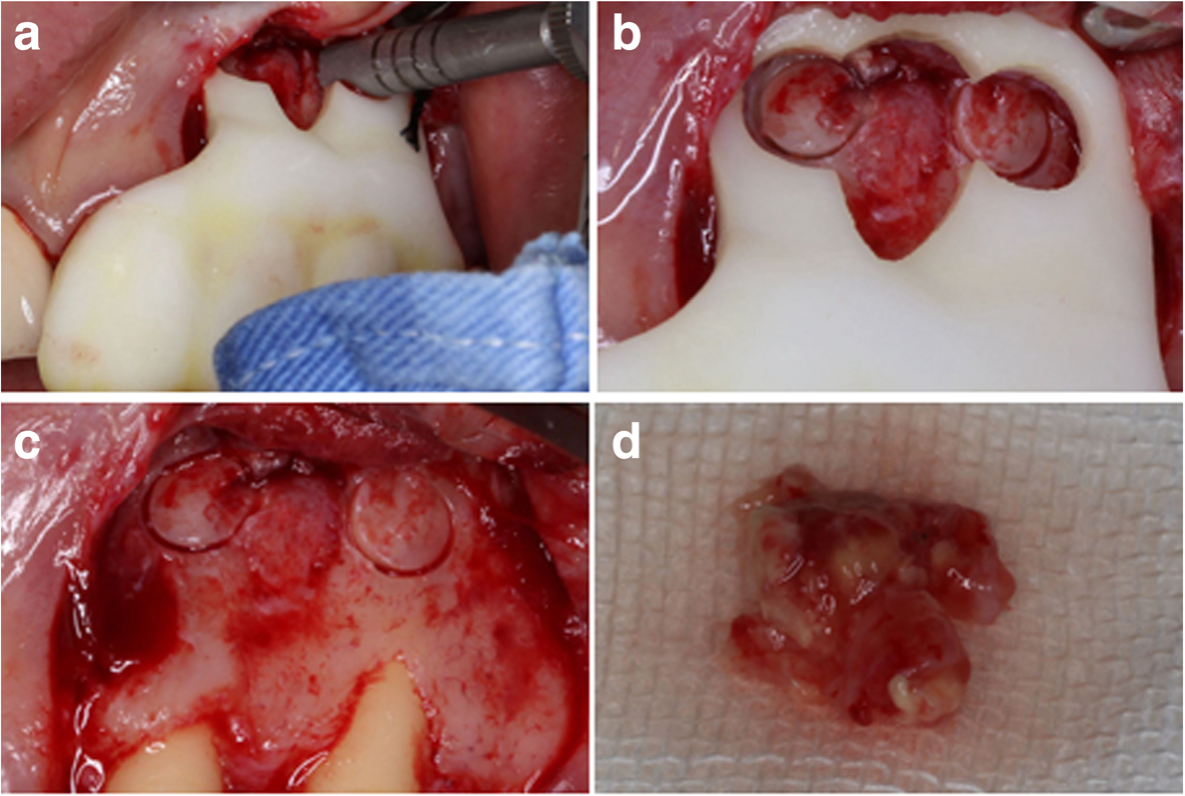
**a** The trephine was positioned. **b** After the trephine reached total working depth I and was removed, the annular notches were observed. **c** The template was removed and the operating site was inspected. **d** The pathological tissues were removed for biopsy

The template was positioned on the teeth and was checked again for stability, a clear operating vision and a straight access to the cortical bone (Fig. 3f). The trephine was laid inside the pre-designed track and was slowly and carefully pushed in with the guidance of the template with constant sterile saline flushing (Fig. 4a). The trephine was removed when it reached total working depth I which was pre-gauged on the CBCT images, leaving an annular notch (Fig. 4b). The template was detached to inspect the operating site (Fig. 4c). The annular cortical bone was gently removed to expose the pathological tissues. The left maxillary lateral incisor and canine were both operated on in the same way, but the total working depth was different in each case. Other soft pathological tissues between the two root-ends were easily removed with suitable sizes of sharp surgical bone curettes (Fig. 4d). The removed pathological tissues were sent for histopathological examination. The template was positioned again after the removal of pathological tissues. The trephine was laid inside and when it reached total working depth II, a sense of dropping was felt through the trephine just as the root-end was separated entirely from the tooth, forming a cutting bevel at the resected root-end perpendicular to the long axis of the canal.

**Fig. 5:**
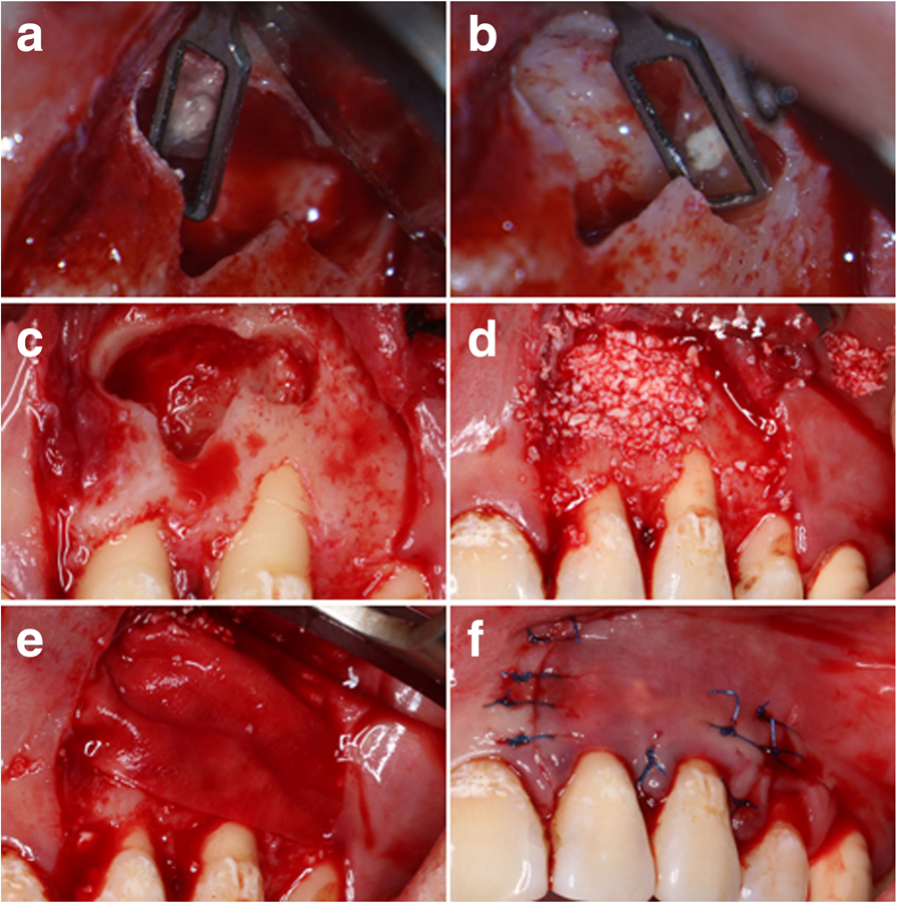
**a**, **b** Micro-surgical mirror was used to examine the cut root surface after the MTA was delivered into the root-end. Completed MTA root-end filling was obtained. **c** After removal and cleaning of soft pathological tissue, the lesion size was large and required a GTR procedure for better prognosis. **d**, **e** Bio-Oss and Bio-Guide were used in this case. **f** The flap was sutured back

**Fig. 6:**
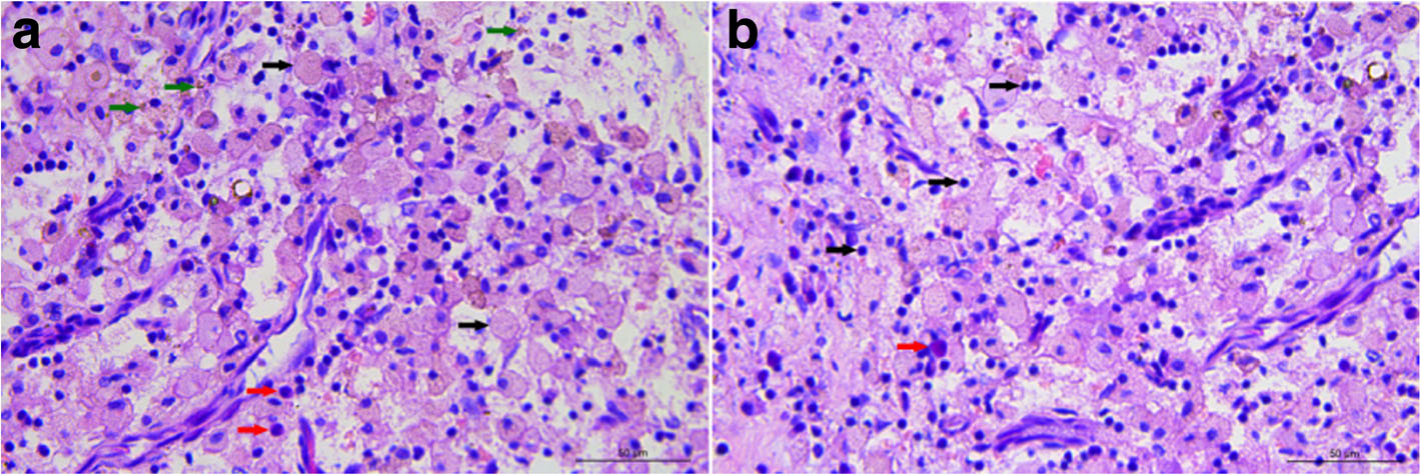
Histopathologic examination revealed the presence of (**a**) eosinophils (red arrows), foam cells (black arrows) and areas of hemosiderin pigmentation (green arrows; H&E, 40×); **b** plasma cell (red arrow) and many lymphocytes (black arrows; H&E, 40×). The features were consistent with periapical granuloma

Root-end cavity preparation was carried out using an angled micro-surgical ultrasonic tip under a microscope. The root-end cavity was prepared, cleaned and dried. Mineral trioxide aggregate (MTA) was filled into the cavity (Fig. 5a and b). Considering the large size of the lesion (Fig. 5c), a guided tissue regeneration (GTR) procedure was adopted for better healing. It was carried out using xenogeneic bone (Geistlich Bio-Oss, Switzerland) and collagen membrane (Geistlich Bio-Gide, Switzerland; Fig. 5d and e). The flap was gently eased back and sutured (Fig. 5f). Pressure was applied for ten minutes after suturing. Biopsy findings were periapical granuloma (Fig. 6a and b).

**Fig. 7:**
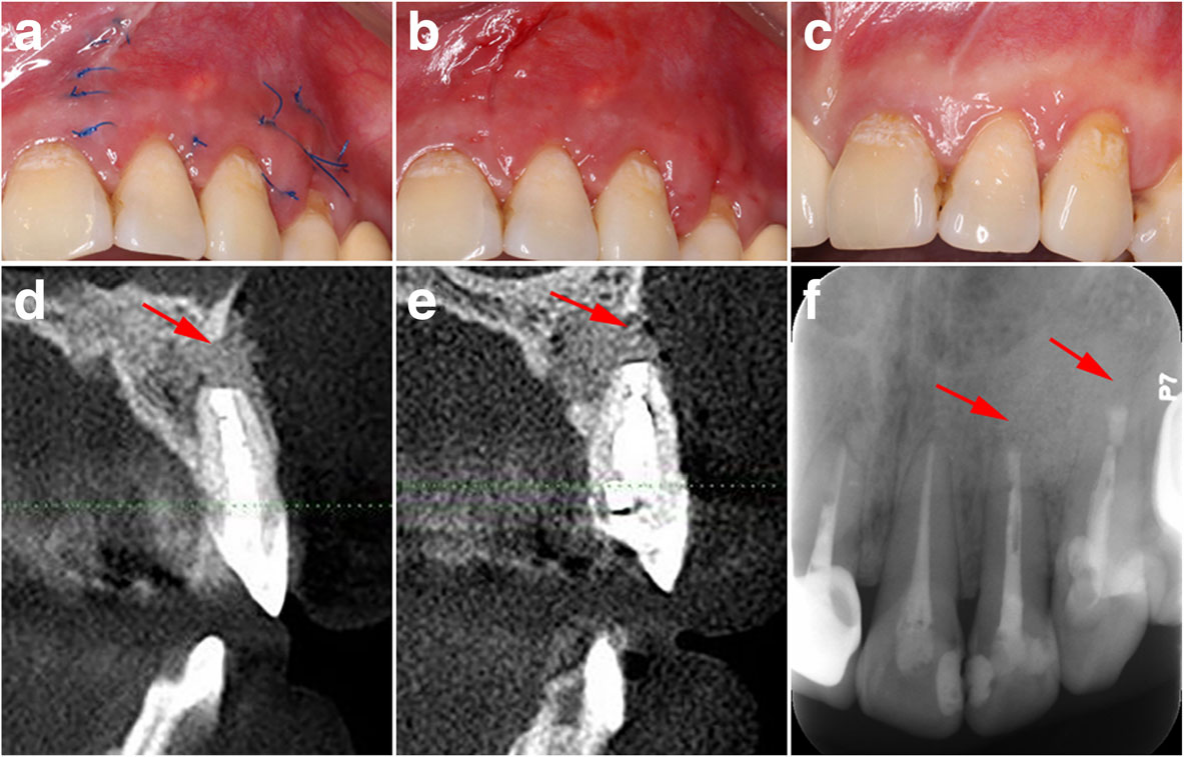
**a** A 7-day review to remove the sutures. **b** The mucosa at the operating site was healing well. **c** The incisions healed well at a six-month review. **d** A sagittal section from a six-month review CBCT showed evidence of bony healing of the left maxillary lateral incisor. **e** The same was true of the left maxillary canine. **f** One-year follow up radiographic examination showed complete healing of the periapical lesion of both teeth and no periapical radiolucency was observed. The red arrow indicates the surgical site

The patient was reviewed 7 days later to remove stitches. The operating site was healing well, and no unusual symptoms or postoperative discomforts were reported by the patient (Fig. 7a and b).

A six-month review showed evidence of bony healing and both teeth were symptom-free (Fig. 7c, d and e). One year after the surgery, the patient was asymptomatic clinically and showed complete bony healing. No periapical radiolucency was observed on radiographic examination (Fig. 7f).

## Discussion and conclusions

Endodontic surgery is needed for the treatment of a large, cyst-like periapical lesion [1, 2]. Sometimes it is challenging to locate root-end for resection [4]. The length of resection of root-end (3 mm) is not easy to control for inexperienced surgeons. Here, a novel method was used to solve these problems with the guide of a 3D-printed template. This template was fabricated following data acquisition, image processing and manufacturing, through which the combined information obtained from CBCT and digital surface scans could be integrated into a physical template.

In a conventional periapical surgical process, searching for root-end and the need for adequate operative visual field usually leave a large bone defect which seems unnecessary now [19]. With the aid of the 3D printed template, the diameter of the lesion caused by surgery could be restricted to 3–4 mm, only slightly larger than the length of resection (3 mm). This minimal invasive surgical procedure maximally limits injury to osseous tissues. Less damage to osseous tissues results in less haemorrhage during surgery, less postoperative complications, shorter healing time and better prognosis.

The template served as a carrier, carrying the information of the location of the root-end and the size of the periapical lesion, the orientation and angle of the root and its apex, and the thickness of the cortical bone into the surgical procedure. With the aid of the template, the trephine was navigated into the exact location, and the surgeons did not have to mentally transfer the information to the clinical situation. This procedure enabled the surgeons to precisely remove the overlying bone and the root-end using the trephine. This method not only simplified the surgical procedure but also considerably improved the treatment efficiency. More time was needed preoperatively to virtually design the template. However, the time will surely be reduced in the future once a workflow is established.

Adjacent teeth and bone were saved from accidental damage with the restriction of the template. This procedure eliminated the unpredictability of osteotomy and root end resection, rendering a challenging clinical procedure relatively simple to manage. The 3D technology described has the potential to substitute for the specialized training and/or clinical experience necessary to treat these difficult cases, which would enable many dentists to achieve predictable results without needing extensive surgical skills.

A slight mismatch between planning and execution may be expected if we consider the accuracy of this 3D planning technology. Further studies need to be carried out to confirm the accuracy of the 3D-printed template-aided periapical surgery procedure. The uniformity can be checked by comparing pre and postoperative virtual images.

This procedure still has some limitations. When the lesion is in a posterior region, the template can still be fabricated and positioned, but insufficient space will be available for the trephine. The costs of such 3D planning and the production of the directional template are considered high; however, such costs will be reduced in the future given the fast-paced development of digital technology in dentistry. There is a promising chance that a reasonable therapy workflow will be established and this treatment approach will be applied in daily routine practice, benefiting more patients.

The digitally designed directional template fabricated using CBCT, CAD and 3D printing technology worked in all aspects to facilitate the periapical surgery as anticipated. The root-ends were accurately located using the template and resected with the trephine. The surgical procedure was simplified, and the treatment efficiency was improved. This technique minimized the damage to soft and hard tissues and reduced iatrogenic injury.

## Abbreviations

3D: three-dimensional
CAD: Computer aided design
CBCT: Cone-beam computed tomography
DICOM: Digital imaging and communications in medicine
GTR: Guided tissue regeneration
MTA: Mineral trioxide aggregate
STL: Stereolithography
